# Synthesis and Characterization of Orange Peel Modified Hydrogels as Efficient Adsorbents for Methylene Blue (MB)

**DOI:** 10.3390/polym15020277

**Published:** 2023-01-05

**Authors:** Saedah R. Al-Mhyawi, Nader Abdel-Hamed Abdel-Tawab, Rasha M. El Nashar

**Affiliations:** 1 Department of Chemistry, College of Science, University of Jeddah, Jeddah 22233, Saudi Arabia; 2 Chemistry Department, Faculty of Science, Cairo University, Giza 12613, Egypt

**Keywords:** adsorbent hydrogel, orange peel, methylene blue, poly acrylic acid hydrogel, cationic dyes

## Abstract

In recent years, due to the developments in the textile industry, water contaminated with synthetic dyes such as methylene blue (MB) has become an environmental threat based on the possible impacts in terms of chemical and biochemical demand, which leads to disturbance in aquatic plants photosynthesis, besides their possible toxicity and carcinogenicity for humans. In this work, an adsorbent hydrogel is prepared via free radical polymerization comprising acrylic acid (PAA) as a monomer and orange peel (OP) as a natural modifier rich in OH and COOH present in its cellulose and pectin content. The resulting hydrogels were optimized in terms of the content of OP and the number of cross-linkers and characterized morphologically using Scanning electron microscopy. Furthermore, BET analysis was used to follow the variation in the porosity and in terms of the surface area of the modified hydrogel. The adsorption behavior was found to follow pseudo-second-order as a kinetic model, and Langmuir, Freundlich, and Temkin isotherm models. The combination of OP and PAA has sharply enhanced the adsorption percent of the hydrogel to reach 84% at the first 10 min of incubation with an adsorption capacity of more than 1.93 gm/gm. Due to its low value of pHPZc, the desorption of MB was efficiently performed at pH 2 using HCl, and the desorbed OP-PAA were found to be reusable up to ten times without a decrease in their efficiency. Accordingly, OP-PAA hydrogel represents a promising efficient, cost-effective, and environmentally friendly adsorbent for MB as a model cationic dye that can be applied for the treatment of contaminated waters.

## 1. Introduction

Clean water availability for drinking has been a highly important health challenge in recent years. Due to the rapid expansion in industrial effluents from the textile and petrochemical industries, environmental disturbances and pollution problems have highly emerged, affecting the quality of drinking water due to their high content of dyes and toxic substances, urging the need for finding simple and cost-effective remedies to remove such contaminants. Industrial dyes represent one of the most common classes of chemicals that cause drinking water contamination [1]. Based on their charge in aqueous solutions, dyes can be classified into cationic (basic dyes), anionic (acidic dyes), and non-ionic (dispersed dyes) [2,3]. Dyes are mostly applied in the textile industry because of their bright color, water-solubility, low cost, and feasibility of application to the fabric. Yet, these dyes can be released as environmental pollutants not only during the tanning process but also as part of the waste resulting from household laundry drainage water. Due to their aromatic nature, synthetic dye degradation in the environment may lead to the production of highly toxic products that are resistant to oxidizing agents and heat or biodegradation, rendering them to be hazardous not only to humans but also to aquatic life [4].

MB is one of the common cationic industrial dyes that is highly used in cotton, silk, and paper dyeing [5,6]. It also has some applicability in biomedical and therapeutic fields [7] and in food production [8,9]. Many human health diseases were found to result from contact with MB, such as mental disorders and blindness [10,11]. The community of biological systems is also affected as a result of a decrease in the transmittance of sunlight due to MB solubility in water due to the change in its color [12,13]. MB is non-biodegradable, carcinogenic, and toxic; this requires an effective, low-cost, fast, and eco-friendly approach for its removal from water. Several methods, such as liquid–liquid extraction [14], nanofiltration [15,16], coagulation [17,18], ultrafiltration [19,20], adsorption/biosorption [21,22], etc., were reported in the literature.

Among previously reported water treatment methods, adsorption-based methods are considered highly efficient, fast, and inexpensive approaches [23]. “Natural solid bio-adsorbents, especially from plant wastes, are considered to be an attractive remedy for water treatment being eco-friendly, low cost, biodegradable, and reliable in terms of green chemistry friendly materials. Their absorption efficiency depends on several parameters, including the surface area of the adsorbent, particle size, and amount, besides some other interaction conditions such as pH, temperature, and contact time with the target samples.

Many examples are reported in literature involving bio-adsorbents for the removal of toxic chemical compounds, heavy metals, and dyes, some of which include wheat and rice [24], remainings of coffee and tea [25,26], leaves and barks of dry trees [27,28,29], powder of saw and coir [30,31,32], and shell of rice [33,34] and natural polymers such as chitosan, lignin and in some cases micro-organisms that were reported to be capable of dyes degradation [25,35].

Hydrogels are materials with 10% of their cconstitutional weight or volume made of water. The existence of hydrophilic groups in the hydrogel network, such as (-NH_2_, -COOH, -OH, CONH_2_, -CONH, and -SO_3_H), is the main reason for their hydrophilicity [36,37,38]. The hydrogel 3D structure formed by the polymeric building blocks can be tailored by different modifiers to regulate the affinity of hydrogels to various target pollutants and ionic contaminants and enhance their adsorption ability [39].

In this work, poly acrylic acid (PAA) was used as a sorbent hydrogel in the presence of orange peel (OP), a natural waste material, as a modifier to improve the removal of the MB (as a model cationic dye) from the water based on the benefit of the components comprising the cell walls of OP which are mainly hemicellulose (11%), cellulose (22), sugar (23%), and pectin (25%) [40]. These natural polymers present in OP were reported to show good adsorption properties towards cationic chemicals such as MB [41,42]. The optimum adsorption conditions, including OP content, pH, temperature, and initial dye concentration and adsorption kinetics models (pseudo-first and pseudo-second-order) and isotherm models (Langmuir, Freundlich, and Temkin), were investigated.

## 2. Materials and Methods

### 2.1. Materials

All chemicals used were of analytical grade and used without further purification. Deionized distilled water was used in preparation of reagents throughout all experiments. Acrylic acid (AA) from Merck Company, Darmstadt, Germany, Ammonium persulfate (APS), ethylene glycol dimethyl acrylate (EGDMA), and boric acid from Alfa Aesar (Kandel, Germany). Nitric acid and sodium Hydroxide from VWR Chemicals (Darmstadt, Germany). Phosphoric acid, sodium nitrate (NaNO_3_), Acetic acid, and Methylene blue (MB) (Sigma-Aldrich company (Schnelldorf, Germany). MB Stock solutions were prepared by dissolving different amounts of powdered dye in Britton–Robinson buffer (BR-B) at different pHs.

### 2.2. Instrumentation

Jenway UV-Visible Spectrophotometer Model 7205 was used for MB initial and final concentration measurements (Jenway Instruments, St Neots, UK). Jenway 3510 Advanced Bench pH Meter (Jenway Instruments, St Neots, UK. Digital orbital shaker, Mini-scale, SSM1, (Stuart, London, UK), was used for shaking the tested samples during the different adsorption stages. An FTIR-Affinity-1, (Shimadzu Corporation, Kyoto, Japan) was used in the range 400–4000 cm^−1^. Ultra-high-resolution scanning electron microscope (SEM) (Model: Leo Supra 55, Zeiss Sigma, Oberkochen, Germany) was used for surface morphology; samples were fixed on aluminum stubs and coated with gold before observation. Brunauer–Emmett–Teller (BET) measurements were used to determine the specific surface area, pore size, and volume using surface area analyzer manufacturer by Quantachrome; model of NOVA touch LX2, the sample was degassed at 150 °C for 2 h under a vacuum.

### 2.3. Preparation of OP Powder

The orange peel used in this study comes from local Egyptian sweet orange fruit (*Citrus sinensis)*. The waste orange peels collected from fresh juice shops or household waste were washed with distilled water, then the colored outer layer of peel was removed by a scrapper to avoid any possible color overlap during the adsorption measurements. The remaining white inner layer of orange peel (OP) was cut into small parts, dried in the microwave, then ground and sieved by a 0.045 mm porous stainless-steel sieve. The resulting dried biomass was stored in a plastic cup until use without any further chemical treatment.

### 2.4. Adsorbent Hydrogel Preparation

In order to prepare OP-PAA absorbent hydrogel, as given in Table 1, different amounts of OP were added to deionized DW in 30 mL screw bottle and sonicated for 10 min, followed by stirring for another 10 min in an oil bath at 65 °C. After complete dispersion of OP, the AA monomer (different ratios) was added while stirring to mix, followed by EGDMA as a cross linker (different ratios). After complete homogeneity of the mixture, it was purged with nitrogen gas for 10 min to remove any entrapped oxygen; finally, 5 mL DW containing 250 mg APS was added to the mixture as an initiator. The polymerization reaction was allowed to take place for 2 h at 65 °C, and the resulting OP-PAA polymers were left to dry in a vacuum oven at 60 °Cand crushed to be ready for further characterization.

### 2.5. Characterization

#### 2.5.1. The Swelling Capacity Percent (SCP)

In order to study OP-PAA swelling behavior, 0.5 g of OP-PAA in a nylon tea bag was added to 50 mL BR-B (pH 4, 7, and 9) at room temperature (RT), followed by orbit shaking at 130 rpm for 3.5 h. The swelled hydrogel was then taken out from the buffer and weighted, then returned to the buffer again at definite time intervals.

The swelling capacity percent (SCP) was calculated according to the following Equation (1)

(1) $$SCP=\frac{W_{f}-W_{i}}{W_{i}}*100\;$$

where $W_{f}$ is the weight of dried OP-PAA, and *W_i_* is the weight of swelling OP-PAA.

#### 2.5.2. Determine the Point of Zero Charges (pHPZC)

The solid addition method was used to determine the point of zero charges (pH_PZC_) of the polymer, according to a previous study [43]. Briefly, 25 mg of OP-PAA was added to 25 mL of 0.1 M NaNO_3_. The pH of sodium nitrate solution was adjusted prior to the addition of OP-PAA (from 2 to 10) using either NaOH or HNO_3_. The solutions were left for 2 days until equilibrium took place, and the pH of each solution was recorded. Δ pH values were calculated and plotted on the x-axis against the pH on the y-axis.

#### 2.5.3. FTIR Spectra, SEM, and BET Characterization

Fourier transform infrared spectra in the range of 4000–400 cm^−1^ were used to characterize the chemical structures of prepared polymer with and without dye. Furthermore, SEM and BET were used to determine polymer morphology and surface area, respectively.

### 2.6. Dye Adsorption and Kinetic Studies

Different OP content by weight (0.5, 1.0, and 2.0 g) of OP-PAA was used to study the OP-PAA hydrogel adsorption efficiency where 250 mg OP-PAA was added to 25 mL MB dye solution (100 mg/L BR-B pH 9.0), and the mixture was agitated at 170 rpm. The dye concentration was measured at different time intervals until equilibrium took place during the kinetic studies. The initial and final concentrations of MB were determined by UV–VIS spectrophotometer and using a linear equation (y = 0.1751x − 0.1145, R^2^ = 0.9998) in the range from 1 to 8 mg/L at λ_max_ 662 nm. The adsorption capacity and removal ratio for the dye can then be determined according to the following Equations (2) and (3):

(2) $$q_{e}=\frac{\left(C_{i}-C_{f}\right)*V}{m}$$

(3) $$RR=\frac{C_{i}-C_{f}}{C_{i}}*100$$

where *q_e_* (mg/g) is the adsorption capacity, *RR* is the removal ratio, *C_i_* (mg/L) is the initial dye concentration, and *C_f_* (mg/L) is the final dye concentration. *V* (L) dye solution volume and *m* (g) represents the dried adsorbent weight.

### 2.7. Desorption Studies

The OP-PAA used in adsorption experiment was separated from the remaining dye by centrifugation, then washed by DW to get rid of unabsorbed MB dye. A total of 2.0 g of Separated adsorbent were then added to 25 mL DW at different pH for 1 h at RT.

## 3. Results and Discussion

### 3.1. Characterization of OP-PAA Hydrogel

#### 3.1.1. The Swelling Capacity Percent (SCP)

The swelling behavior of a hydrogel adsorbent plays an important role in its adsorption characteristic [44]. Nine hydrogel polymers (A-I) were prepared with different amounts of AA as a monomer, OP, and EGDMA as a cross-linker, as given in Table 1. The swelling capacity percent (SCP) was determined for the prepared polymers to select the best component ratio exhibiting the highest swelling capacity.

**Figure 1:**
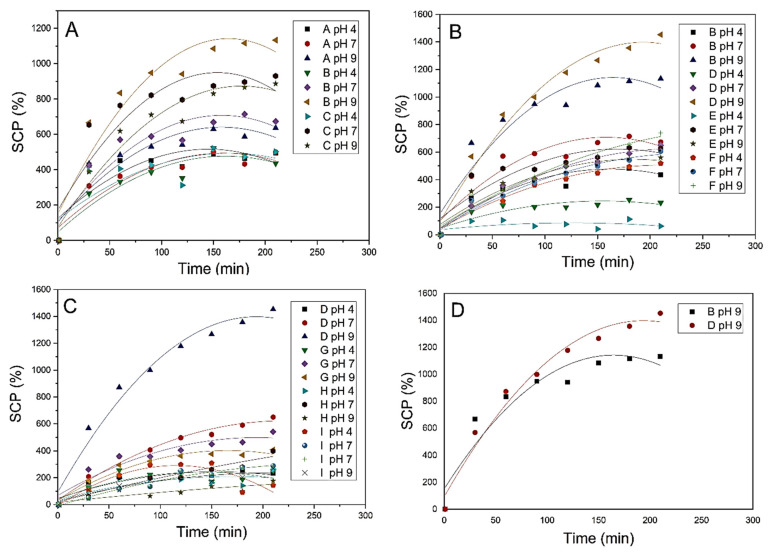
Effect of AA (**A**), OP (**B**) and EGDMA percent on SCP (**C**), and comparison between polymers (**B**,**D**) exhibiting the highest absorption efficiency (**D**).

Figure 1A shows the effect of AA percent on the swelling capacity at different pH values. It is clear that the increase in AA percent from 10% (polymer A) to 20% (polymer B) led to an increase in SCP. This increase was due to an increase in the content of carboxylic acid groups. On further increase in AA content from 20% (polymer B) to 30% (polymer C) caused a decrease in SCP. This phenomenon can be attributed to the interaction between the OH groups of OP and the COOH group in AA, rendering the polymer more rigid and, in turn, affecting its swelling tendency [45,46,47].

Figure 1B represents the effect of variation of OP content effect on SCP. The addition of 0.5 g OP (polymer D) causes an increase in SCP due to introducing more ionizable groups such as OH and COOH present in the cellulose and pectin content of OP, respectively. Further increase in OP to 1 or 2 g was found to cause deterioration of SCP; as a result, the viscosity increased due to the high content of OP, which hinders interaction of the adsorbents with MB [48].

Finally, the effect of using EGDMA as a cross-linker was studied, as given in Figure 1C. It is clear that the increase in EGDMA concentration was associated with SCP decrease because it renders the water diffusion in the compact hydrogel more difficult. The tendency of SCP to decrease with cross-linker concentration increase was found to be in agreement with the Flory theory and the results of previous studies [49].

The pH effect on SCP was also investigated, and it was found that an increase in SCP was associated with a pH increase. According to the previous result, a hydrogel adsorbent polymer composed of 6 mL AA and 0.5 g OP without using a cross-linker (polymer D) was selected to be used for further adsorption experiments.

#### 3.1.2. Determine the Point of Zero Charges (pHPZC)

**Figure 2:**
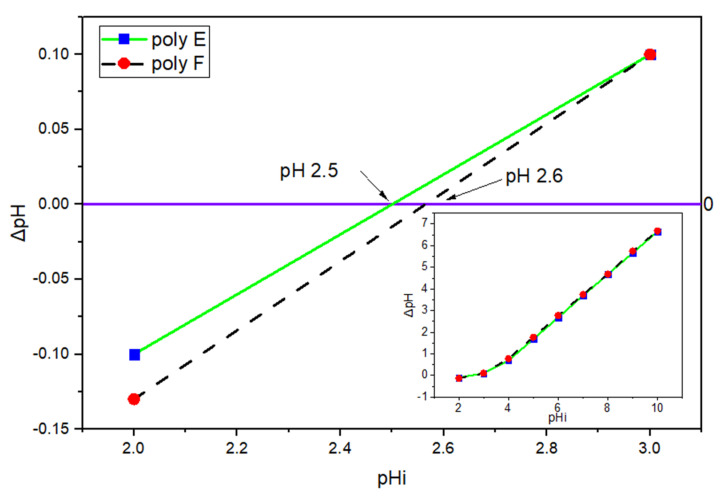
Relation between ΔpH and initial pH and pH_PZC_ conclusion.

pH_PZC_ value plays an important role in the adsorption mechanism understanding where adsorption of cationic dyes such as MB is preferred to take part at pH larger than pH_PZC_ [50]. From the experimental data, it was found that pH_PZC_ values of PAA and poly E are 2.6 and 2.5, respectively, as indicated in Figure 2.

#### 3.1.3. FT-IR Spectra

**Figure 3:**
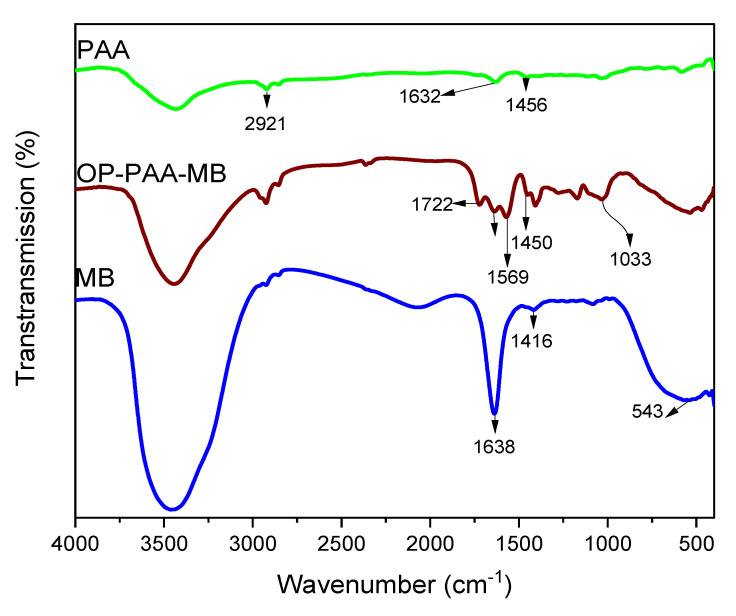
FT-IR spectra of PAA, MB, and MB adsorbed on polymer E hydrogel.

The FT-IR spectra of PAA, MB, and MB adsorbed on OP-PAA are shown in Figure 3. In PAA FT-IR spectra, a band at 2921 cm^−1^ that refers to the C-H stretching of an alkane is noticed due to complete AA polymerization. Another two bands at 1632 and 1456 cm^−1^ can be assigned to symmetric and asymmetric stretching vibrations of COO-, respectively. FT-IR spectrum of MB has many bands; the most characteristic are 1638, 1416, and 543 cm^−1^, which correspond to C=N stretching, C–N stretching vibration in aromatic amines, and C–S skeleton vibration, respectively [51,52].

Due to OP’s complex nature, a lot of bands were noticed in its FTIR spectrum. The bands 1722, 1033, and 1450 cm^−1^ can be associated with C=O stretching, indicating the abundant carboxylic groups that are capable of interaction and binding with MB; also, the band at 1569 cm^−1^ can be assigned to N-O stretching.

The FT-IR spectra of PAA, MB, and MB adsorbed on OP-PAA showed a clear OH band at 3500 cm^−1^, the reduction in the intensity of the OH band indicated its participation in blending and cross-linking mechanism with the cationic MB dye, which agrees with the previously reported results for orange peel interaction with textile dyes indicating the role of OH groups in adsorption [53,54].

#### 3.1.4. BET

**Figure 4:**
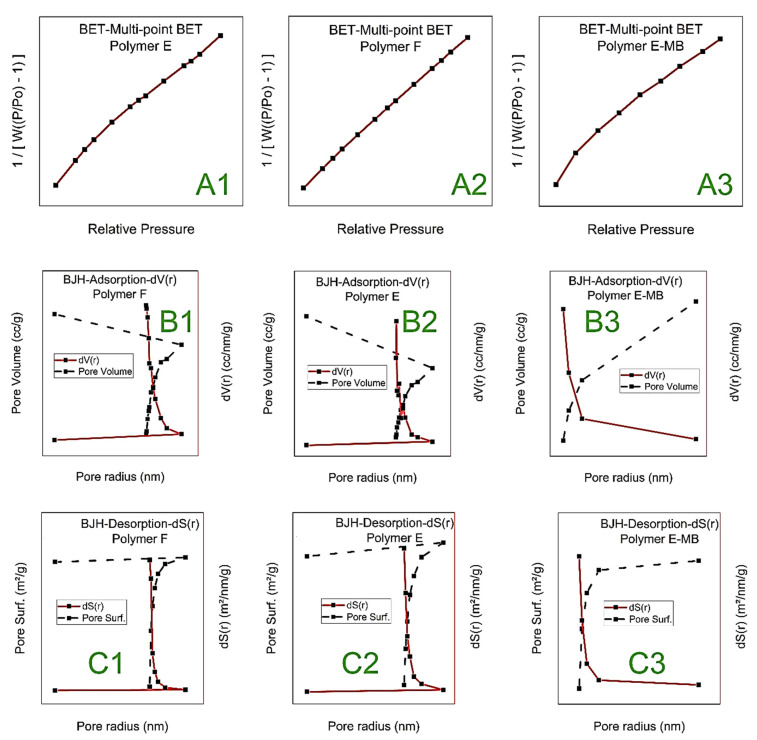
BET-Multi-point BET for polymer F (**A1**), polymer E (**A2**), and MB adsorbed on polymer E (**A3**). BJH Pore Size Distribution-Adsorption for polymer F (**B1**), polymer E (**B2**), and MB adsorbed on polymer E (**B3**). BJH Pore Size Distribution-Desorption for polymer F (**C1**), polymer E (**C2**), and MB adsorbed on polymer E (**C3**).

The surface area (SA) of the adsorbent is one of the main influencing parameters affecting its adsorption efficiency. Multipoint Brunaur–Emmett Teller (BET) analysis was used to determine the polymer’s SA using N_2_ gas adsorption and desorption, as given in Figure 4A. While, Barrett–Joyner–Halenda (BJH) analysis was used to detect the pore volume of polymers F, E, and polymer E after adsorption of MB (E-MB) were found to be 0.090 cc/g, 0.111 cc/g, and 0.025 cc/g, respectively as given in Table 2 and Figure 4B,C. Polymer F was found to show a relatively higher surface area (SA) of 66.5 m^2^/g compared to that of polymer E 53.1 m^2^/g and polymer E-MB 22.9 m^2^/g. Although the SA of polymer E is smaller [45] than that of polymer F, the adsorption capacity of polymer E was found to be the largest.

These phenomena can be correlated to the large pore volume of polymer E and its high content of OH and COOH groups as a result of OP addition. Polymer E-MB has the smallest pore volume and SA due to the adsorption of MB dye on the polymer surface. Furthermore, the pore radius of polymer E was found to decrease from 1.734 nm to 1.684 nm in the presence of MB; this can be attributed to the high adsorbed amount of MB, which indicated the strong interaction between the OP and the PAA polymer [45,46,53].

#### 3.1.5. SEM

**Figure 5:**
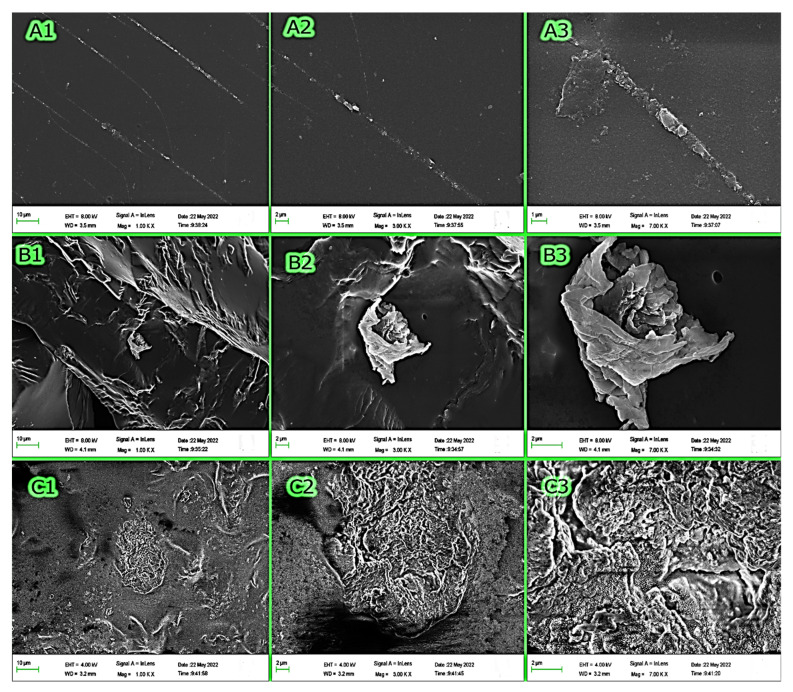
SEM images of polymer F (**A**), Polymer E (**B**), and MB adsorbed on polymer E (**C**).

Figure 5 shows the surface morphology of polymer F (5A), polymer E (5B), and polymer E after adsorption of MB (E-MB) (5C) at three different magnification power (1, 3, and 7 KX). Polymer F, comprising PAA only, showed a homogenous polymeric nature, as shown in Figure 5A, whereas the addition of OP to PAA, Figure 5B, was found to increase the surface area by increasing the pores and cavities in the polymeric matrix. The change in textural properties and roughness of polymer E after the adsorption of MB, as shown in Figure 5C, represented evidence of the accumulation of MB onto the bio-adsorbent surface.

### 3.2. Effect of OP Content

**Figure 6:**
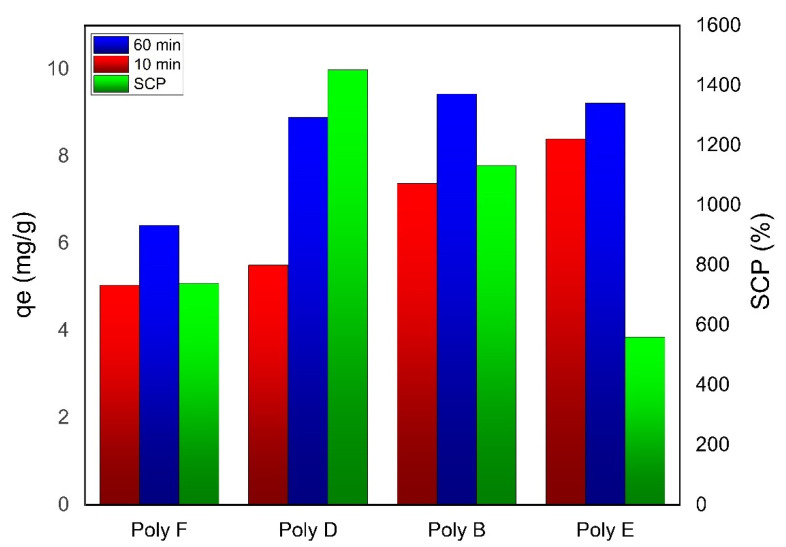
Effect of OP content on q_e_ of both PAA and OP-PAA for MB adsorption. Experimental conditions were: MB concentration 100 mg/L; adsorbent hydrogel dose 250 mg in 25 mL; pH 9 at RT.

The effect of OP content on swelling and hydrogel adsorption capacity is presented in Figure 6. It is clear that upon the addition of 0.5 g OP to PAA, Polymer D, q_e_ was increased compared to polymer F comprising PAA only. This can be attributed to the introduction of OH and COOH groups from cellulose and pectin, respectively, that represented the main components in OP, where –OH reacts with AA and increases the polymeric network [55,56]. The increase in the swelling capacity also participates in adsorption capacity enhancement due to the increase in the surface area of the adsorbent [44,57,58].

Further increase in the content of OP, though, increases the adsorption capacity and velocity of adsorption, but on the other hand, results in a decrease in the swelling efficiency. This can be explained based on the increase in the number of –OH groups due to the increase in cellulose content, which may act as a cross-linker upon reaction with AA monomer increasing the polymer rigidity. However -COOH groups on the pectin surface are capable of maintaining the adsorption capacity value stable and increasing the rate of adsorption. According to OP content results, polymer E, comprising 6 mL AA and 2.0 g OP, was selected as hydrogel adsorbent to complete the next adsorption experiments as it showed the highest adsorption capacity among the prepared hydrogels.

### 3.3. Effect of pH

**Figure 7:**
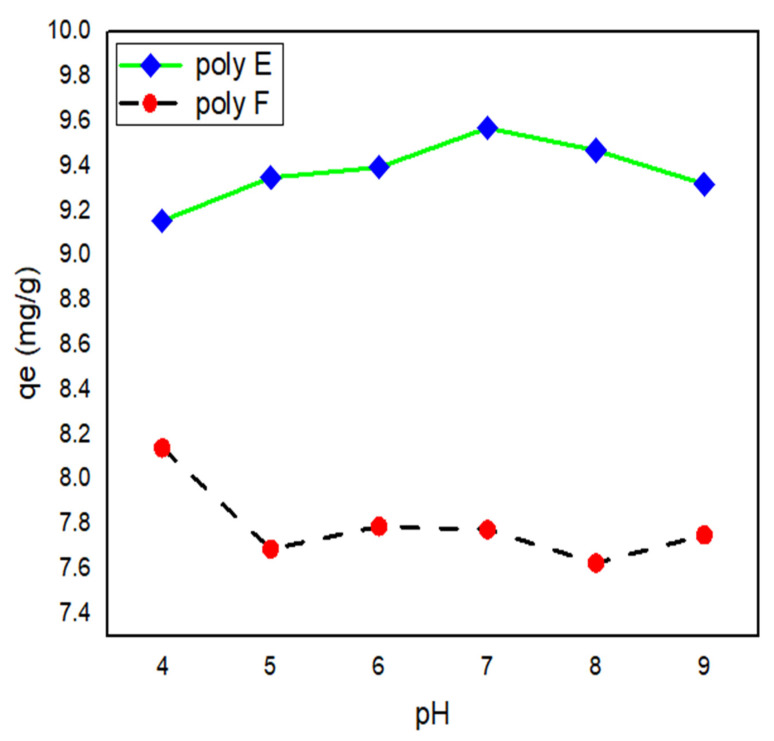
Effect of pH on q_e_ of both polymer F and polymer E for MB adsorption. Experimental conditions were: MB concentration: 100 mg/L; h adsorbents hydrogel dose: 250 mg in 25 mL and RT.

The variation of pH of the medium plays an important role in protonation and deprotonation not only of the MB but the hydrogel adsorbent itself due to its enriched content of carboxylic and hydroxyl groups; thus, the effect of pH on adsorption of MB (100 mg/L) at RT was investigated. In order to discuss the pH effect of MB adsorption, the pH_PZC_ must be determined where cationic dye adsorption is preferred at a pH higher than pH_PZC_ [39,50], as given in Figure 7. From experimental data, pH_PZC_ values of PAA and polymer E were found to be 2.6 and 2.5, respectively.

The variation in q_e_ was found to be relatively small at low pH and peaks at pH 7.0. This can be attributed to the protonation of -COOH at low pH, where -H from HCl is bound to the carboxylic group in acrylate. As the pH increases, COOH becomes deprotonated and more COO- groups are generated and are available for reaction with MB [59]. On the other hand, the increase in pH value results in an increase in the swelling ratio paired with increased polymer surface area and more MB penetration. At pH higher than 7.0, the q_e_ was found to decrease due to the charge screening effect of sodium ions and competition between sodium ions from NaOH and MB to interact with the carboxylate groups of the polymer [60,61].

### 3.4. Effect of Temperature

**Figure 8:**
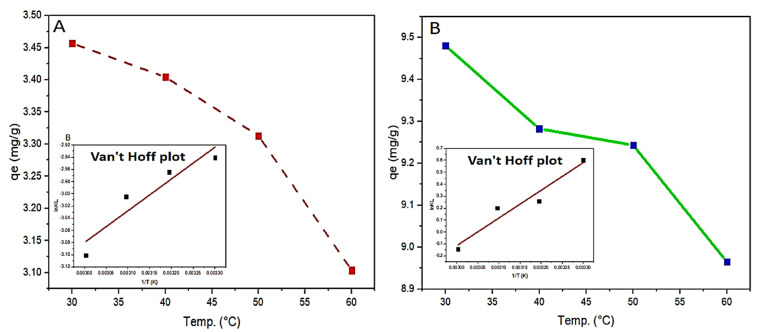
Effect of temperature and Van’t Hoff plot on q_e_ of polymer F (**A**) and polymer E (**B**) for MB adsorption. Experimental conditions were: MB concentration: 100 mg/L; adsorbents hydrogel dose 250 mg in 25 mL; pH 7.

The effect of temperature on adsorption is an important factor to be investigated in order to have an insight into the adsorption thermodynamics parameters such as entropy ΔS, enthalpy ΔH, and free energy ΔG. MB adsorption on PAA and OP-PAA adsorbent hydrogel was found to decrease with a temperature increase from 30 to 60 °C, as shown in Figure 8. Although the increase in temperature commonly causes swelling of internal adsorbent construction and, in turn, increases the dye molecules penetration [62], it also causes dye ions’ mobility to increase. This increase in ion mobility is associated with an adsorption capacity decrease, which agrees with the behavior previously reported [63,64].

The change in entropy ΔS, enthalpy ΔH, and free energy ΔG was calculated by using Equations (4)–(7) [65,66]:

(4) $$K_{d}=\frac{\left[A\left(s\right)\right]}{\left[A\left(l\right)\right]}=\frac{q_{e}}{C_{e}}$$

(5) $$\Delta G=-\mathrm{RT}\ln K_{d}$$

(6) ΔG=ΔH−TΔS

(7) $$\ln\left(K_{d}\right)=\frac{\Delta S}{R}-\frac{\Delta H}{\mathrm{RT}}$$

where C_f_ and C_i_ are the final and initial MB concentrations (mg/L), K_d_, R, and T are the equilibrium constant, the gas constant, and temperature (K), respectively.

Figure 8 represents the Van’t Hoff plot where ln K_d_ was plotted against 1/T. ΔS and ΔH can be calculated from the Van’t Hoff plot using the intercept and slope. Table 3 shows the thermodynamic values; by increasing Temperature, ΔG values move to be a positive value, which means that the MB adsorption process on the hydrogel adsorbent is spontaneous [67,68]. ΔH and ΔS values are negative, as commonly associated with exothermic processes, indicating a randomness decrease at the solid/liquid interface [69,70].

### 3.5. Adsorption Kinetics

**Figure 9:**
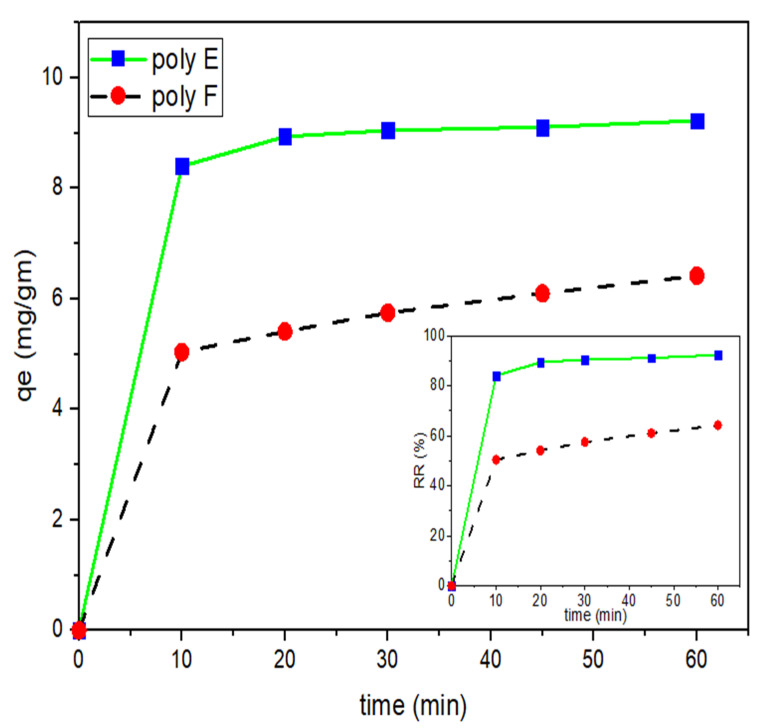
Effect of contact time on q_t_ of both PAA and OP-PAA for MB adsorption. Experimental conditions were: MB concentration 100 mg/L; adsorbents hydrogel dose 250 mg in 25 mL; pH 9 at RT.

In order to investigate the adsorption kinetics of OP-PAA and MB, the time effect on adsorption capacities was studied. Figure 9 shows the relationship between time (min) and polymer E and F adsorption capacities at a definite time (min) and qt (mg/g). It can be noticed that, at the first 10 min, a rapid adsorption rate took place where about 84% of MB was adsorbed by OP-PAA. Then, the rate of adsorption became slow until equilibrium took place at 60 min with a removal ratio, RR, of about 92%). This can be attributed to the presence of a large, uncovered absorbent surface area at the beginning of the experiment, which decreased over time upon the start of MB adsorption and accumulation of the adsorbent surface. On further increase in time for another hour, the RR value was found to reach 94% and remained stable thereafter when tested up to 5 h.

On the other hand, on comparing with unmodified PAA as an adsorbent, RR values were found to be 50% and 64% at 10 and 60 min, respectively, with no further increase. Accordingly, it can be concluded that the addition of OP to PAA enhances the removal ratio from 50% to 84% in the first ten min and increases the RR rate to 92% after one hr.

The adsorption kinetics were tested using both pseudo-first and pseudo-second-order models [71].

The pseudo-first-order linear Equation (8) can be represented as [59]:

(8) $$\log\left(q_{e}-{\;q}_{t}\right)={\log\;q}_{e}-\frac{K_{1\;}t}{2.303}$$

while the pseudo-second-order linear Equation (9) can be indicated by [72]:

(9) $$\frac{t}{q_{t}}=\frac{1}{K_{2}{\;q}_{e}^{2}}+\frac{t}{q_{e}}\;$$

where q_t_ (mg/g) and q_e_ are the adsorption capacities at a definite time (min) and equilibrium, respectively, K_1_ (1/min) and K_2_ (g/mg min^−1^) are the rate constants of adsorption for pseudo-first- and second-order, respectively.

**Figure 10:**
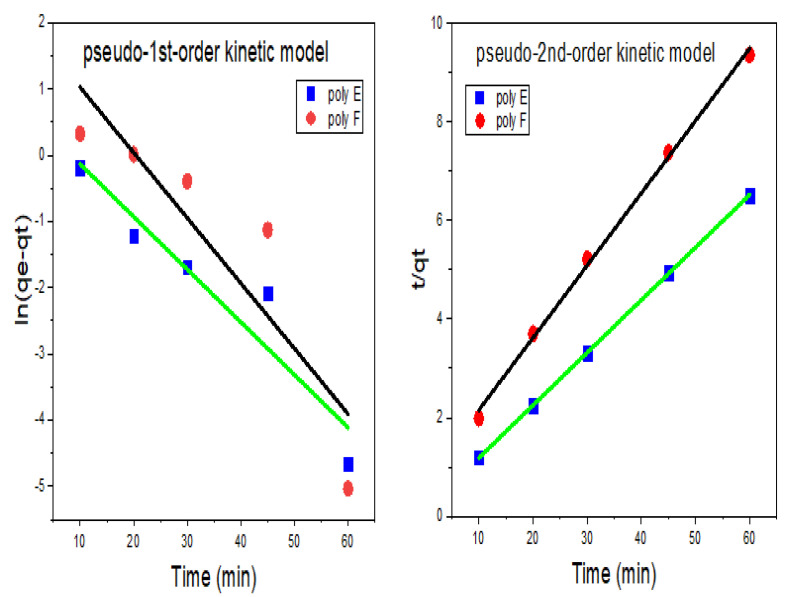
Kinetics curves of pseudo-first-order and pseudo-second-order model. Experimental conditions were, MB concentration 100 mg/L; adsorbents hydrogel dose 250 mg in 25 mL; pH 9; RT.

The experimental data were used to plot $\log\left(q_{e}-q_{t}\right)$ and t/q_t_ against t, as shown in Figure 10, in order to calculate K_1_ and K_2_, and q_e_ from the intercept and slope of the plots as given in Table 4.

R^2^ of the pseudo-first-order model was found to be 0.739 and 0.869 for PAA and OP-PAA, respectively, while the calculated q_e_ were 7.5 and 1.9 mg/g for PAA and OP-PAA, respectively. The previous result indicated that the pseudo-first-order model does not fit with experimental data. In contrast, R^2^ for the pseudo-second-order model was found to be 0.9969 and 0.9999 for PAA and OP-PAA, respectively, indicating better fitting to such a model. Besides the R^2^ value being highly close to 1.0, the calculated q_e_ was close enough to the experimental q_e_; thus, based on the data shown in Table 4, it can be concluded that the pseudo-second-order model is suitable to describe the adsorption of MB onto OP-PAA hydrogel.

### 3.6. Adsorption Isotherms

**Figure 11:**
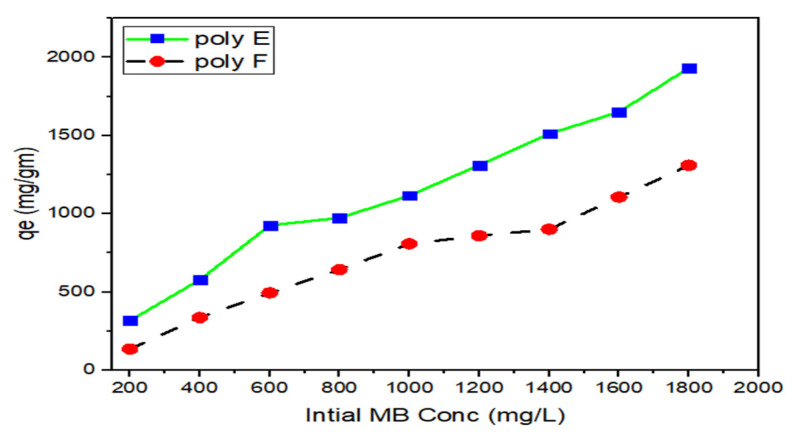
Effect of initial MB concentration on both polymer F and polymer E for MB adsorption. Experimental conditions were: adsorbents hydrogel dose 50 mg in 100 mL; pH 7, and RT.

The correlation between the initial concentration of MB (C_i_) and the equilibrium absorption capacity, q_e_, was studied, as shown in Figure 11. It was found that the increase in C_i_ from 200 to 1800 mg/L led to a dramatic increase in qe from 135 to 1309 mg/g for polymer F and from 317 to 1933 mg/g for polymer E. From this result, it can be concluded that the addition of OP to PAA improves the adsorption efficiency of the adsorbent hydrogel.

Langmuir, Freundlich, and Temkin isotherm models were used to test the interaction mechanism between MB and adsorbent hydrogel.

The Langmuir linear Equation (10) form is [73]:

(10) $$\frac{1}{q_{e}}=\frac{1}{q_{m}}+\frac{1}{q_{m}K_{L}}\frac{1}{C_{e}}$$

The Freundlich linear Equation (11) form is [74]:

(11) $$\log q_{e}={\;\mathrm{logK}}_{f}+\frac{1}{n}{\mathrm{logC}}_{e}$$

The Temkin linear Equation (12) form is [23]:

(12) $$q_{e}=\frac{\mathrm{RT}}{b}\;\ln\left(K_{T}C_{e}\right)$$

where q_m_ (mg/g) and qe represent the maximum and equilibrium adsorption capacities, respectively. C_i_ (mg/L) is the initial dye concentration, while K_L_ (L mg^1^), K_F_ (mg^1−1/n^ L^1/n^ g^−1^), and K_T_ Lg^−1^ are the isotherm constants of Langmuir, Freundlich, and Temkin, respectively.

**Figure 12:**
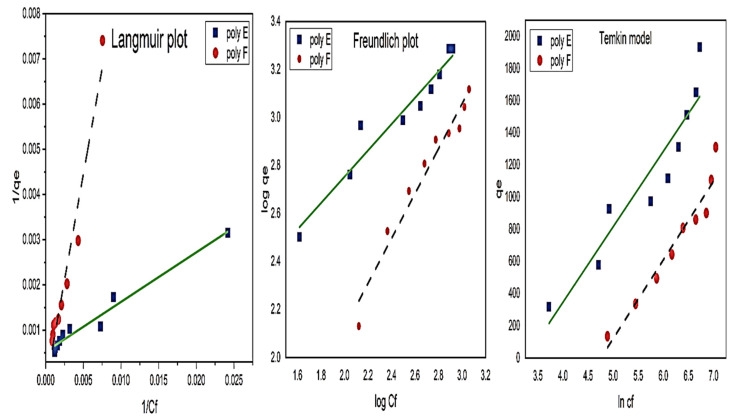
Isotherm model plots of both polymer F and E for MB adsorption. Experimental conditions were: adsorbents hydrogel dose 50 mg in 100 mL; pH 7, at RT.

Figure 12 and Table 5 show the fitting curves of the tested isotherm models and their parameters. The highest R^2^ value for polymer E was found to be related to the Langmuir isotherm model (0.964) with a calculated q_m_ close enough to the experimental qe. Freundlich and Temkin’s models showed R^2^ of 0.932 and 0.873, respectively. This indicated that the adsorption of MB on polymer E could be better described by Langmuir rather than Freundlich and Temkin models.

On the other hand, R^2^ of Langmuir and Freundlich for polymer F were equal (0.95) with negative sign q_m_ when calculated using Langmuir models, which indicated that MB adsorption on polymer F follows Freundlich rather than Langmuir’s model. Furthermore, the Temkin model showed a good R^2^ value (0.929) and can be used to describe the adsorption of MB on polymer F.

### 3.7. Desorption of MB

In order to study the regeneration and reusability of the OP-PAA, the polymers bound to MB were separated by centrifugation, then washed by DW to get rid of any unabsorbed MB dye. A total of 2.0 g of the separated adsorbent were incubated in 25 mL DW of different pH values for 1 h at RT. Desorption studies play a role in understanding the adsorption mechanism.

It is noteworthy to mention that if the MB adsorbed on the hydrogel adsorbent can be removed by DW, this means that the attraction between MB and hydrogel adsorbent is very weak. On the other hand, if the attraction between MB and hydrogel adsorbent can be destroyed by an acid such as HCl, this indicates that the mechanism can be correlated more to electrostatic attraction or ion exchange [50].

**Figure 13:**
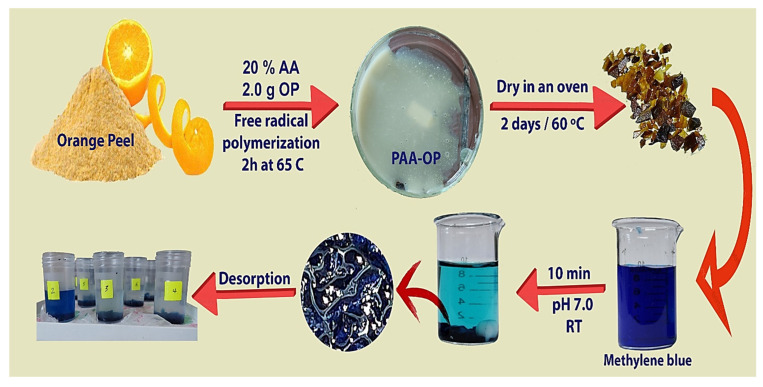
Adsorption and Desorption of MB from Polymer E at different pH (2–9). Experimental conditions were: adsorbents hydrogel dose: 2.0 g in 25 mL for 1 h at RT.

The experimental results showed the occurrence of no desorption of MB from the hydrogel adsorbent at pH ranging from three to nine, while efficient removal of MB was attained at pH 2, as represented in Figure 13. Desorption at pH 2 can be correlated to the low value of pH_PZC_ of the hydrogel adsorbent and indicated that the MB was attached to the hydrogel adsorbent via electrostatic attraction rather than surface adsorption.

The desorbed OP-PAA hydrogel reusability was tested, and it was found to retain its efficiency in terms of absorption capacity without any change for not less than ten times of usage and desorption, which indicated the usability of the hydrogel polymer after simple treatment with HCl.

## 4. Conclusions

In this work, a simple approach for the removal of cationic dye based on the combination of poly acrylic acid (PAA) as a sorbent hydrogel in the presence of Orange peel (OP) as a natural modifier to improve the removal of the MB (as a model cationic dye) is reported. MB adsorption by OP-PAA and PAA was found to be affected by the amount of OP, initial pH value, reaction temperature, and initial dye concentration. The addition of OP to PAA was found to enhance the adsorption capacity of PAA and accelerate the adsorption process. q_e_ of both polymer E and F adsorbent hydrogels were optimized at pH 7, and RT temperature increases were found to have a negative effect on q_e_. The max adsorption capacity of polymer E was calculated by the Langmuir isotherm to be equal to 1892 mg/g.

Comparison of the adsorption parameters of OP-PAA hydrogel adsorbent, presented in this work and other previously reported dyes’ adsorbents given in Table 6 [75,76,77,78,79,80,81,82], indicated that the presented adsorbent is not only an inexpensive and cost-effective, green and recyclable but was found to be more effective and fast acting for dye removal, where 84% of the adsorption was attained in 10 min without any need for stirring. The desorption process of MB, as a model cationic molecule, from OP-PAA hydrogel adsorbent was found to efficiently take place at pH 2.0 indicating that MB interaction with the hydrogel adsorbent took place via electrostatic attraction rather than surface adsorption which indicates the efficient ability of the hydrogel to retain the adsorbed dye particles. Furthermore, the prepared OP-PAA adsorbent hydrogel was found to be regenerable using HCl at pH 2 and retained its efficiency after several adsorption/desorption cycles, which indicated that the presented OP-PAA adsorbent hydrogel is a promising cost-effective, and efficient, reusable eco-friendly adsorbent for water treatment, from cationic moieties that can either be dyes or heavy metal ions.

## Figures and Tables

**Table 1:** OP-PAA Adsorbent hydrogel composite component.

Polymer Name	AA (%)	AA (mL)	OP (g)	EGDMA (mL)	DW (mL)
**A**	10	3.0	1.0	---	22.0
**B**	20	6.0	1.0	---	19.0
**C**	30	9.0	1.0	---	16.0
**D**	20	6.0	0.5	---	19.0
**E**	20	6.0	2.0	---	19.0
**F**	20	6.0	---	---	19.0
**G**	20	6.0	0.5	0.6	18.4
**H**	20	6.0	0.5	1.2	17.8
**I**	20	6.0	0.5	1.8	17.2

**Table 2:** Surface area, pore volume and radius of polymer F, polymer E, and MB adsorbed on polymer E.

	Unit	Polymer F	Polymer E	Polymer E-MB
Surface Area by multipoint BET	mc²/g	66.51	53.11	22.86
Pore Volume by BJH	cc/g	0.090	0.111	0.025
pore radius by BJH	nm	2.087	1.734	1.684

**Table 3:** Polymers E and F thermodynamic parameters for MB adsorption.

	Temperature	Polymer E	Polymer F
ΔG (KJmol^−1^)	30 °C	−1.52	7.41
	40 °C	−0.67	7.71
	50 °C	−0.54	8.07
	60 °C	0.40	8.59
ΔH (KJmol^−1^)		−19.23	−4.34
ΔS (KJ^−1^mol^−1^)		−58.64	−38.62

**Table 4:** Polymer E and F kinetic parameters for MB adsorption.

	Pseudo-First-Order				Pseudo-Second-Order			
Parameters	K_1_ min^−1^	Cal q_e_ mg/g	Exp q_e_ mg/g	R_1_^2^	K_2_ mg^−1^ min^−1^	Cal q_e_ mg/g	Exp q_e_ mg/g	R_2_^2^
Polymer E	−0.0013275	1.9	9.2	0.8692	0.09582068	9.4	9.2	0.9999
Polymer F	−0.0016448	7.5	6.4	0.7384	0.03129885	6.8	6.4	0.9969

**Table 5:** Polymers E and F isotherm parameters for MB adsorption at 25 °C.

	Parameters	Unit	Polymer E	Polymer F
Langmuir model	q_m_	mg/g	1892	−4379
	K_L_	L/mg	0.00482	−0.00024
	R^2^		0.964	0.953
Freundlich model	1/n		0.5485	0.9357
	K_f_		45.3	1.78
	R^2^		0.932	0.950
Temkin model	B_T_	J/mol	469	489
	K_T_	L/gm	0.0383	0.0086
	R^2^		0.873	0.929

**Table 6:** Comparison between OP-PAA and some other previously reported dyes’ adsorbents.

Adsorbent	Dye	q_m_ mg/g	RR%	Time	Ref
Tragacanth gum and carboxyl-functionalized carbon nanotube	Methylene blue	1092	80.0	40 min	[75]
Sludge	Direct red 28	1.25	---	100 min	[76]
*Haloxylon recurvum* stem	Acid brown 354	6.87	81.0	50 min	[77]
Coconut Shell (Activated carbon)	Crystal violet	44.00	99.6	24 h	[78]
Orange peel	Methylene blue	---	95.7	24 h	[54]
Spent tea leave	Methylene blue	---	99.0	24 h	[54]
Rattan sawdust	Methylene blue	359.00	---	480 min	[79]
Acacia nilotica sawdust	Methylene blue	46.95	99.9	60 min	[80]
Starch-g-poly (acrylic acid)	Methylene blue	1532	----	30 min	[81]
Gum ghatti-g-poly(acrylic acid)	Methylene blue	909	99	75 min	[82]
Orange peel poly (Acrylic acid)	Methylene blue	1892	84.0	10 min	**This work**

## Data Availability

All data will be available upon reasonable request from the corresponding author.
